# All bets are on: obsession, engagement, and moral tension in sports betting behavior

**DOI:** 10.3389/fpsyg.2025.1608414

**Published:** 2025-07-14

**Authors:** Ronald A. Yaros

**Affiliations:** Philip Merrill College of Journalism, University of Maryland, College Park, MD, United States

**Keywords:** sports betting, fan engagement, obsession, gambling behavior, NFL, uses and gratifications theory, moral concern, digital media consumption

## Abstract

**Introduction:**

As legalized sports betting becomes increasingly integrated into American sports culture—particularly within the NFL—concerns have emerged about how such practices influence fans’ emotional engagement, behavioral dependency, and moral judgment. This study investigates age-based variation in betting behaviors and identifies key predictors of media consumption, perceived dependency, and concern for others’ gambling habits.

**Methods:**

A cross-sectional online survey (*N*= 492) was administered in 2024 using snowball sampling to recruit NFL fans. Branching logic ensured that only those with direct sports betting experience completed all attitudinal questions. Fifteen Likert-scale items measured cognitive, behavioral, and emotional responses to betting. Exploratory factor analysis (EFA) identified latent constructs, and multiple regression models tested four research questions related to fan engagement, moral concern, dependency, and intervention attitudes.

**Results:**

EFA revealed four dimensions: Personal Betting Habits, Betting-Driven Enjoyment, Concern for Others, and Perceived Social Addiction. Perceived obsession with betting was the only consistent and strong predictor of increased sports media engagement. Demographic factors such as age and gender were not significant predictors. Concern for others’ gambling behavior was only weakly related to personal dependency and confrontation attitudes.

**Discussion:**

Findings suggest a form of cognitive dissonance among sports bettors, who may recognize problematic behavior in others but refrain from acting on it, especially when they are highly engaged themselves. This study contributes a multidimensional model of betting-related engagement and highlights the need for improved psychometrics, stakeholder awareness, and future longitudinal research on the psychological complexities of sports gambling.

## Introduction

Sports betting has emerged as a dominant force within the global gaming industry, driven by rapid technological innovation, mobile platforms, and increasing media coverage. This growth has reshaped the cultural and psychological landscape of sports fandom. Once limited to periodic wagers placed in person, sports betting is now a continuous, pervasive activity available with just a few taps on a phone. Recent research highlights that betting behavior, particularly sports betting, is driven by overlapping social, emotional, and financial motivations (Lee et al., 2024). These include the thrill of potential rewards, peer competition, and heightened emotional investment in the outcomes of sports.

In the United States, the 2018 Supreme Court ruling striking down the Professional and Amateur Sports Protection Act (PASPA) enabled states to legalize sports betting, accelerating the industry’s integration into mainstream sports culture. Since then, more than 30 states and Washington, DC. have legalized sports gambling. During a 2025 CBS *Sunday Morning* segment, veteran journalist Ted Koppel reported that just a few days of March Madness betting alone were projected to generate $3 billion in wagers. In total, the U. S. sports betting market exceeds $200 billion in annual value (Holden, 2019). Leading platforms include *FanDuel*, *DraftKings*, *BetMGM*, *PointsBet*, and *Caesars Sportsbook*. Globally, more than 30,000 sports-betting-related businesses contribute to an industry that continues to expand in both size and influence.

Despite growing concern, many fans continue to view betting as an exciting enhancement to their sports experience. In 2024, NFL games made up 72 of the 100 most-watched U. S. television broadcasts, and some analysts credit betting with expanding the league’s audience, particularly among younger male viewers (Adgate, 2025). Digital sports media—from journalism sites to team content and betting apps—have increasingly incorporated multimedia elements, such as live odds, podcasts, and social media integrations, to sustain user attention. These trends illustrate how sports betting is closely tied to digital engagement strategies aimed at capitalizing on fleeting user interest.

Sports betting has not only grown in scale but in intensity. Scholars note that it has shifted from a discontinuous to a continuous form of gambling, characterized by increased availability, higher betting frequency, and more dynamic wagering options such as in-play or micro-bets (Lopez-Gonzalez and Griffiths, 2018). This transformation raises new psychosocial concerns. Research shows that sports betting, especially among younger adults and students, can detract from academic performance, strain social relationships, and contribute to anxiety, depression, and financial instability (Avenyo et al., 2024; Shipurut and Dauda, 2024).

College campuses have become an especially lucrative target for betting companies. Students often engage in sports betting not only for entertainment but also as a normalized leisure activity (Dannemiller et al., 2025). At the same time, a meta-analysis of 65 studies spanning over a decade found consistent psychosocial risks associated with sports betting across international contexts (Etuk et al., 2022). In Europe, online sports betting accounts for over one-third of the gambling market, with “cash-out” options and other in-play features increasing user involvement (Killick and Griffiths, 2018). Nations also differ in regulatory responses. China and Korea maintain stricter oversight, while Australia, the UK, and the U. S. tend to favor commercial growth. This global convergence of digital gambling, sports entertainment, and data marketing has created complex challenges for public health and policymakers.

Several studies have identified risk factors linked to problematic betting behavior. These include ease of access, targeted advertising, and live in-play betting features that can amplify one’s impulsivity and diminish control (Kim et al., 2023; Parke and Parke, 2019). In-play betting has been associated with greater financial harm and emotional volatility, especially when combined with alcohol or drug use (Hing et al., 2016; Russell et al., 2019). Similarly, Wilkins et al. (2024) found that increased participation in fantasy sports, often linked to sports betting platforms, correlates with more frequent and intense emotional responses, underscoring the deep psychological engagement that many users experience.

Still, key gaps remain in understanding how sports betting influences broader patterns of fan behavior, emotional attachment, and moral judgment. Much of the existing research focuses either on economic outcomes or on pathological gambling, with limited attention to the spectrum of everyday engagement and the psychosocial factors that may distinguish casual bettors from more dependent users. The present study addresses this gap by examining how fans engage with NFL football in relation to their sports betting behavior and demographic profile.

This study situates betting behavior within a broader framework of digital engagement (Sundar and Limperos, 2013), recognizing that sports fans today interact with multimedia platforms in ways that blur the lines between consumption, participation, and emotional immersion.

Despite the explosion of research into gambling disorders and addiction, relatively little empirical work has examined how normative, non-pathological betting behavior shapes patterns of digital fan engagement, emotional response, and moral perception. Four research questions guided this study:

RQ1: Do people report watching more sports or consuming more sports media because of betting?

RQ2: How do emotional and moral concerns about sports betting relate to engagement and dependency?

RQ3: What individual characteristics predict greater dependency and fear of missing out (FOMO) associated with sports betting?

RQ4: What predicts the belief that individuals should confront others about their sports betting behavior?

These questions are explored through a survey of participants who responded to general perception items about sports betting. Participants who reported personal betting experience or awareness of others’ betting behavior were routed to more detailed questions assessing betting habits and emotional reactions. The study aims to reveal the psychosocial and cognitive patterns that may underlie problematic or emotionally dependent betting behavior.

### Theoretical framework

This study draws upon two major theoretical frameworks to contextualize the relationship between sports betting and digital fan engagement: Uses and Gratifications Theory (UGT) and the Theory of Planned Behavior (TPB).

Uses and Gratifications Theory proposes that media users are active participants who seek out content that satisfies their psychological or social needs (Katz et al., 1973; Ruggiero, 2000). In the realm of sports betting, fans may turn to wagering platforms not simply for economic reward but to amplify suspense, increase their emotional connection to the game, and fulfill social needs. Placing a bet transforms viewing into a participatory experience, reinforcing excitement and engagement (Lole et al., 2019; Lopez-Gonzalez and Griffiths, 2018). Betting platforms also offer gamified interfaces, personalized statistics, and community features, making them well-suited for satisfying individual and interpersonal gratifications (Thomas et al., 2012).

The Theory of Planned Behavior (Ajzen, 1991; Ajzen et al., 2018) provides additional insight into the psychological mechanisms that drive betting behavior. According to the theory, the intention to engage in a behavior is shaped by attitudes toward the behavior, perceived social norms, and perceived behavioral control. In the context of sports betting, fans with positive attitudes (e.g., belief that betting is fun or profitable) are more likely to engage in betting. Similarly, if a fan’s social networks endorse gambling, the normative pressure to participate increases. Fans who believe they can control their betting behavior, even if they cannot, are more likely to justify continued participation (Clark, 2010).

Prior research suggests that young, male, educated individuals, often full-time students or employees, are at heightened risk for problematic gambling (Hing et al., 2023). Social norms, especially in male-dominated sports environments, can also glamorize betting as a competitive or masculine pursuit. Fast-paced in-play betting and fantasy sports intensify the illusion of control, while cultural and technological shifts normalize these behaviors through marketing, social media, and peer influence.

By integrating these two well-documented theories, the present study conceptualizes betting not merely as a behavior but as a form of digital engagement, shaped by individual gratifications, group norms, and perceived agency. It further explores how age and gender act as moderators, influencing how fans interpret the risks and rewards of betting.

## Method

### Sample

Participants (*N* = 492) were recruited using a snowball sampling method through the *Qualtrics* platform. Recruitment was initiated by a class of journalism students who distributed the survey via social media platforms (e.g., X, Facebook, and Instagram) and popular sports fan forums. Participants were asked to share the link with their personal networks to ensure a broader reach across demographic groups. Participation, which followed IRB-approved consent, was voluntary and anonymous. Completion time averaged between 5 and 10 min.

### Survey design and procedure

The survey was designed to measure sports betting behaviors, attitudes, and demographic characteristics, particularly among individuals who engage in sports betting.

The survey included 24 total questions: two demographic items (age and gender), 10 general perception and screening items, and 12 additional Likert-scale items targeting cognitive, behavioral, and emotional betting responses. Fifteen of these Likert-style items were used in the exploratory factor and regression analyses. All participants were asked to respond to the first 12 general items about their perceptions of sports betting.

All participants were asked to respond to the first 12 general items about their perceptions of sports betting. Item 12 functioned as a filter asking: *“Have you placed a bet on a sports game (not including a party-related bet like squares or any bets without money or items of value at stake)?”* Those who answered “No” were directed to the end of the survey. Only participants who responded “Yes” proceeded to the remaining questions designed to capture cognitive, emotional, and behavioral aspects of the sports betting experience. This ensured that the analytic sample reflected self-identified sports bettors.

#### Measures

Key betting-related items included,

*“Watching games with the betting connection makes the sport more enjoyable.”*

*“I only watch games if I am betting on them.”*

*“I like to place bets on games I do not watch.”*

*“I feel that I am ‘missing out’ if I do not place a bet on a game I am watching.”*

*“I often feel obsessed with sports betting.”*

Participants exposed to the AddictionCenter.com’s definition of betting addiction as *“a behavioral disorder characterized by a persistent and uncontrollable desire to bet on sports despite adverse outcomes,”* were asked, “How many people do you think are obsessed/addicted to sports betting?” Those knowing at least one person responded to “I feel concerned about family/friends I consider obsessed with sports betting” and “I feel it is important for me and others concerned about a family member/friend to confront them about their betting habits”.

### Data analysis

Data were analyzed using IBM SPSS Statistics (version 29). Exploratory factor analysis (EFA) was conducted on 15 Likert-scale items using principal components extraction and Varimax rotation to identify underlying latent constructs. Factor reliability was evaluated using *Cronbach’s alpha*. Based on EFA results, four composite factors were identified: Personal Betting Habits, Betting-Driven Enjoyment, Concern for Others, and Perceived Social Addiction. Reliability was assessed using Cronbach’s alpha or inter-item correlations, depending on the number of items per factor.

Subsequent analyses included independent-samples t-tests to assess age differences, bivariate correlations to examine relationships between key variables, and three hierarchical multiple regressions to identify predictors of sports engagement, dependency, and confrontation attitudes. Regression models were limited to participants who indicated betting behaviors, reducing the analytic sample for regression analyses to 305 participants. This reduction was due to survey branching (non-bettors were excluded from key items) and listwise deletion of cases with missing values on predictor or outcome variables.

## Results

An exploratory factor analysis (EFA) was conducted using 15 Likert-scale items that measured participants’ attitudes, behaviors, and concerns related to sports betting. The goal was to identify underlying dimensions of sports betting engagement and perception among NFL fans. Before extraction, sampling adequacy was assessed. The Kaiser-Meyer-Olkin (KMO) measure of sampling adequacy was 0.780, indicating middling to meritorious suitability for factor analysis. Bartlett’s Test of Sphericity was significant, *χ*^2^ (105) = 781.33, *p* < 0.001, confirming that the correlation matrix was not an identity matrix.

**Figure 1:**
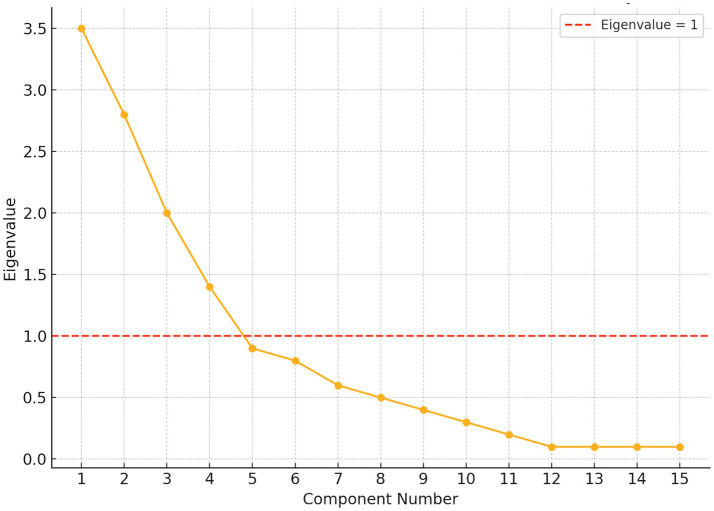
Scree plot of eigenvalues from factor analysis.

Principal components extraction with Varimax rotation revealed a four-factor solution, accounting for 44.88% of the total variance. Communalities ranged from 0.30 to 0.69, supporting acceptable levels of shared variance between items and extracted components. The scree plot and eigenvalues >1 criterion supported the four-factor solution, which converged after seven iterations (Figure 1). Factor interpretation was based on thematic coherence of item loadings (threshold ≥ 0.40), yielding the following dimensions:

Factor 1: Personal Betting Habits (e.g., “I feel obsessed with sports betting” and “I feel that I am missing out if I do not bet on a game”).

Factor 2: Betting-Driven Enjoyment (e.g., “I watch games not involving my favorite team because of betting” and “Watching games with the betting connection makes the sport more enjoyable.”)

Factor 3: Concern for Others (e.g., “I am concerned about family/friends I consider obsessed with sports betting” and “I enjoy watching sports less because of betting”).

Factor 4: Perceived Social Addiction (e.g., “Based on your opinion, how many people do you know who are obsessed with sports betting?”)

Factors 1 and 2 each included five items, Factor 3 included two items, and Factor 4 was composed of two perceived social observation items. Internal consistency was evaluated using Cronbach’s alpha for multi-item factors and inter-item correlation for two-item factors: Factor 1: *α* = 0.619, Factor 2: *α* = 0.502, Factor 3: *r* = 0.223, Factor 4: *r* = 0.244.

**Figure 2:**
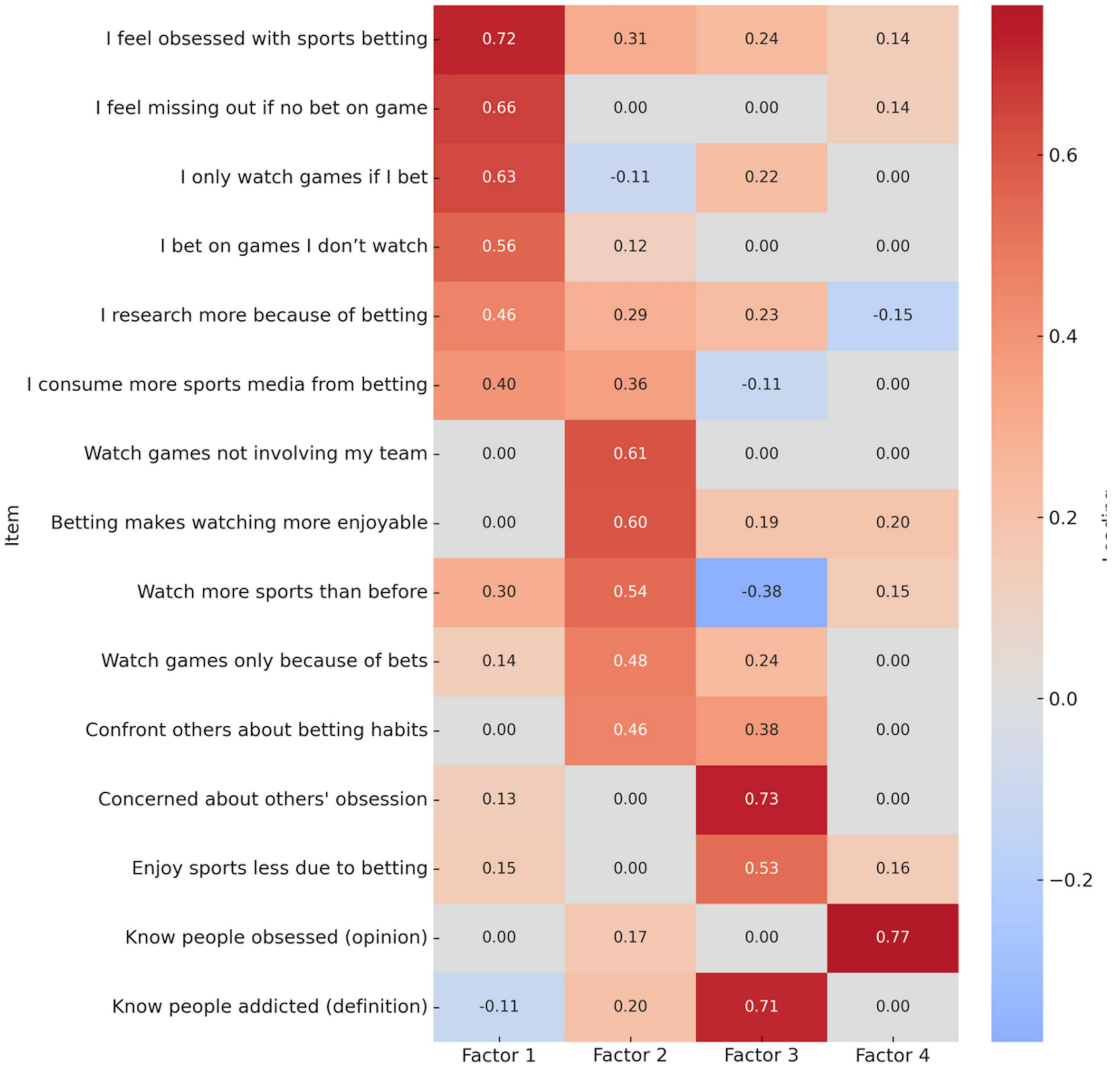
Component loading matrix for sports betting items.

While Factor 2’s alpha was marginal, the factor was retained due to theoretical importance and acceptable item coherence. A heatmap of item loadings across the four components is shown in Figure 2.

The first research question asked whether sports betting increases sports media engagement? Descriptive statistics were calculated for the full sample (*N* = 493), including participant demographics and mean scores for each factor. Participants ranged in age from 18 to 64 (*M* = 41.25, SD = 13.35). 245 identified as female, 248 as male. Personal betting habits for males (*M* = 3.22, SD = 1.48) were almost identical to those of females (*M* = 3.21, SD = 1.49), as were Betting-Driven Enjoyment: males (*M* = 3.32), females (*M* = 3.28).

Age Group Comparisons (Younger: 18–35; Older: 36–64) were not statistically different for Personal Betting Habits*: t* = −0.27, *p* = 0.791 or Betting-Driven Enjoyment: *t* = 0.18, *p* = 0.858.

In a follow-up comparison, the sample was dichotomized using the mean age of 41.25 years. This yielded two groups: a younger group (ages 18–35; *n* = 256) and an older group (ages 36 and above; *n* = 237). An independent-samples t-test revealed that younger participants reported significantly greater Betting-Driven Enjoyment (*M* = 3.57, SD = 0.94) than older participants (*M* = 2.93, SD = 1.06), *t* (491) = 6.37, *p* < 0.001. However, no significant age differences were observed for Bet-Driven Engagement (*p* = 0.788), Concern about others (*p* = 0.295), or Social Addiction/FOMO (*p* = 0.988). These findings reinforce that while younger adults derive more enjoyment from sports betting, they are not necessarily more engaged or more likely to experience concern or dependency.

A multiple linear regression (*N* = 305) was conducted to predict Personal Betting Habits using age, gender, Concern About Others, and Social Addiction/FOMO. The model was approached significance: *F* (4, 300) = 2.35, *p* = 0.055, *R^2^* ≈ 0.03. Age: *β* = 0.0003, *p* = 0.958, Gender: *β* = −0.079, *p* = 0.571, Concern about others: *β* = 0.083, *p* = 0.078 (marginal), and Social Addiction/FOMO: *β* = 0.115, *p* = 0.251.

A second regression (*N* = 439) used expanded attitudinal variables to predict engagement: *F* (8, 430) = 13.26, *p* < 0.001, *R^2^* = 0.198. The only significant predictor was Obsessed: β = 0.73,

*p* < 0.001 Other variables (e.g., Research_Because_Betting, Only_Watch_If_Betting, Social Addiction/FOMO) were not significant, confirming that perceived obsession with betting was the dominant driver of sports engagement.

The second research question asked whether emotional or moral concerns were associated with sports engagement and dependency. Pearson correlations revealed: Factor 1 with Factor 2: *r* = 0.365, Factor 1 with Factor 3: *r* = 0.141, Factor 2 with Factor 3: *r* = 0.185, and Factor 3 with Factor 4: *r* = 0.109. These suggest modest relationships between betting enjoyment, engagement, and concern for others. Regression results, however, showed only marginal prediction from Concern about others.

**Figure 3:**
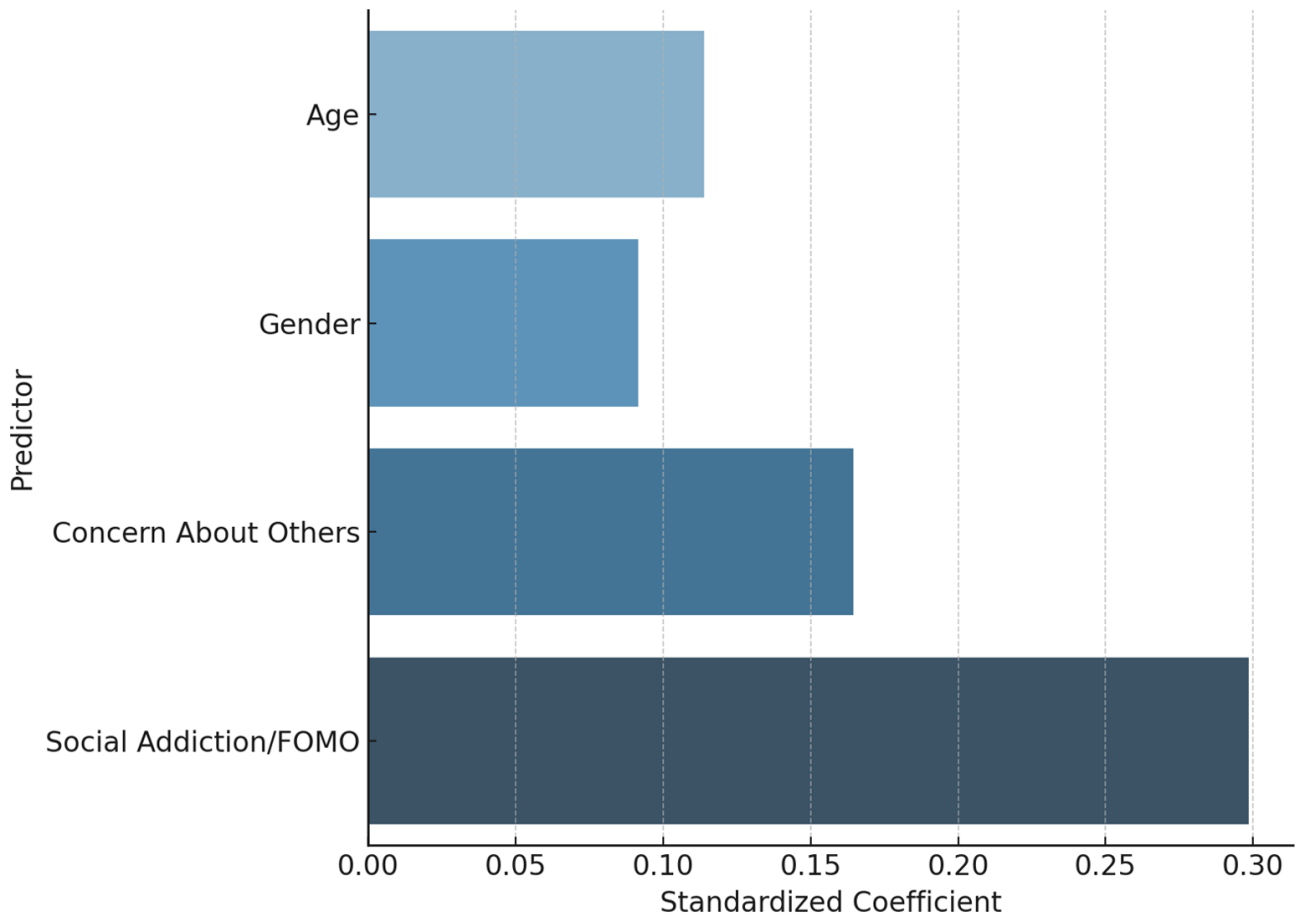
Standardized regression coefficients from model 1.

Figure 3 presents the standardized regression coefficients, visually highlighting that only Concern About Others approached statistical significance, while age, gender, and social addiction were not significant predictors.

**Figure 4:**
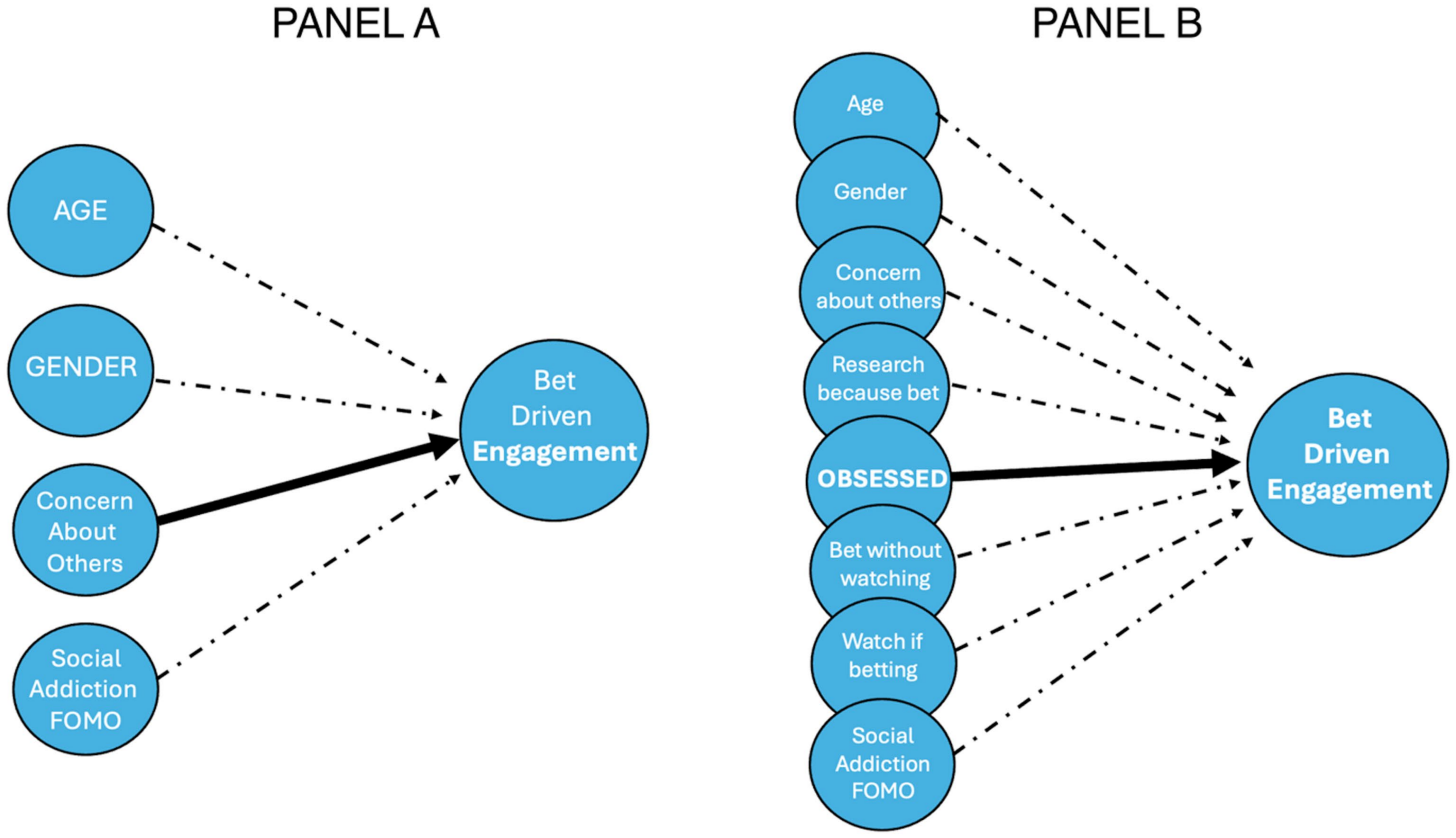
Comparison of conceptual models. Panel A, significant predictors and Panel B, obsession as a sole driver.

Figure 4 illustrates the conceptual models of engagement. Panel A represents Model 1, in which none of the theoretical predictors reached conventional significance thresholds. Panel B illustrates Model 2, where “Obsessed” emerged as the sole significant driver.

These visualizations demonstrate that while emotional and moral concern may co-occur with betting engagement, their direct impact is weak, possibly indicating a dissociation between personal involvement and concern for others.

Research question three asked, what individual characteristics predict greater dependency and fear of missing out (FOMO)? Although dependency was not modeled independently, Factor 1 scores (Personal Betting Habits) served as a proxy. Age and gender were not significant predictors. A marginal trend suggested that older participants scored higher on Perceived Social Addiction (*M* = 2.15 vs. 2.03), *t* = −1.79, *p* = 0.074.

Regression analyses indicated that the best predictor of betting-driven engagement was self-reported obsession, not age, FOMO, or concern for others. These findings suggest that dependency may be self-recognized more than externally attributed.

The final research question (4) asked what predicts the belief that others should be confronted about their betting behavior? While confrontation was not directly modeled, Concern for Others (Factor 3) served as a proxy. Although Concern was positively correlated with other engagement factors, it was not a significant predictor in the regression models. This suggests a disconnect between personal engagement and the willingness to confront others. Individuals who engage in betting may experience cognitive dissonance, acknowledging concern while not endorsing intervention, which may reflect a process of normalization or moral disengagement. Together, these results suggest a nuanced, multidimensional structure of sports betting behaviors, where engagement, obsession, concern, and perceived social risk operate as overlapping yet distinct cognitive domains.

## Discussion

This study addressed four key research questions related to the behavioral, emotional, and perceptual effects of sports betting among NFL fans. RQ1 received the strongest support. Bet-Driven Sports Engagement emerged as a reliable construct through exploratory factor analysis, and regression results clearly identified “obsession with betting” as the dominant predictor of increased sports media consumption. Other predictors, such as age, gender, concern for others, or fear of missing out, did not significantly contribute to explaining engagement (see Figure 1).

Despite popular reports associating sports betting engagement with younger audiences, our regression analysis found no significant predictive effect of age. This suggests that while younger users may engage differently, the psychological marker of *obsession*, rather than chronological age, better explains elevated betting behavior in our sample.

The enhanced regression model reinforced this outcome by showing that only the “Obsessed” variable predicted engagement (see Figure 2B). This finding supports Uses and Gratifications Theory by illustrating how betting provides emotional arousal and personal relevance, thereby fueling greater involvement with sports content.

Although regression analyses showed that age was not a significant predictor of betting-driven engagement, the supplemental *t*-test analysis using the sample mean as a cutoff revealed that younger participants reported significantly higher levels of betting-driven enjoyment. This suggests that age may still influence the affective appeal of betting (e.g., enjoyment), even if it does not directly predict behavioral engagement or dependency. This nuance refines our understanding of age-related patterns and supports more targeted future research into how emotional gratification varies across demographic segments.

Research question 2 investigated the relationship between emotional and moral concerns and engagement or dependency. While correlations revealed modest links between concern and engagement, regression results showed no significant predictive power from moral concern or social addiction. This suggests that individuals who report concern for others’ betting behaviors may not act on those concerns, potentially due to emotional dissonance or social normalization. Despite the theoretical appeal of moral tension influencing betting behavior, the data indicate that such concerns often co-occur with—but do not cause—increased engagement.

The third research question focused on predicting dependency and FOMO. These constructs were indirectly evaluated through scores on the Personal Betting Habits factor and select regression variables. Contrary to expectations, dependency was not significantly predicted by age, gender, or fear of missing out. However, perceived obsession proved to be a strong behavioral marker of dependency. The lack of demographic predictors suggests that psychological rather than structural characteristics drive problematic betting behavior. This pattern hints at self-reinforcing cycles consistent with cognitive dissonance and addictive reinforcement models.

Finally, the fourth research question asked what predicts the belief in confronting others about their betting behavior. Concern for Others was correlated with that belief but did not predict engagement or dependency. Regression findings suggest that individuals with higher betting engagement or obsession are less likely to endorse confrontation, perhaps reflecting moral disengagement or denial. This psychological distancing may help explain why some heavily involved fans fail to recognize or act upon problematic behavior in themselves or others.

The proxy measures used to assess moral judgment, namely “concern for others” and “perceived importance of confrontation,” offer only a partial picture of moral tension. Future work should draw from moral psychology to incorporate validated constructs such as moral disengagement (Bandura, 1999, 2002), guilt proneness (Tangney et al., 1992), or bystander intervention models (Latané and Darley, 1970). These could better capture the psychological barriers that prevent fans from acting on concerns, despite recognizing problematic behavior in others”.

Taken together, these results lend support to a multidimensional model of betting-related behavior. While betting increases media engagement, emotional concern and perceived social risk do not consistently shape behavior. Instead, self-reported obsession is the clearest indicator of elevated engagement, as shown in both regression models and the conceptual path diagram (Figure 4).

### Limitations and directions for future research

Several limitations should be acknowledged when interpreting these findings. First, a cross-sectional design can limit causal inference. While regression models show associations, only longitudinal studies can determine whether engagement leads to obsession or vice versa. The sample size decreased from 492 to 439 due to incomplete responses. Although necessary for statistical integrity, this may reduce generalizability. There is a possible self-report bias. Responses may reflect social desirability, particularly on sensitive items such as obsession or concern.

Future studies should employ longitudinal panel designs to assess whether increased betting activity predicts subsequent changes in engagement, or vice versa. Experience sampling methods (ESM) could capture real-time fluctuations in betting behavior and emotional engagement across sports seasons. Experimental designs might manipulate exposure to betting stimuli (e.g., live odds, social betting cues) to observe changes in arousal, perceived control, or moral disengagement. Such methods could provide stronger evidence for causal pathways, particularly those linking self-reported obsession to emotional reinforcement and disengagement mechanisms.

It is also important to interpret these findings in light of the psychometric limitations observed. While the four-factor solution was supported by exploratory analysis and aligned with theoretical expectations, the internal consistency of Factor 2 (*α* = 0.502) and the two-item measures (*r* = 0.223, *r* = 0.244) fell below conventional reliability thresholds. These scores suggest potential measurement error and underscore the need for future scale development. Specifically, confirmatory factor analysis with larger and more representative samples would help assess the robustness of these constructs. The inclusion of additional items could also enhance internal consistency and conceptual clarity.

The use of snowball sampling initiated through student distribution networks may have skewed the sample toward more digitally engaged users or sports enthusiasts, potentially underrepresenting casual bettors or non-digital gamblers. As a result, levels of self-reported obsession or emotional attachment may be higher than in the general population. Future studies should strive for stratified random sampling or platform-based recruitment to ensure broader representation of the sports betting spectrum.

Although the demographic spread was broad, the sample may not represent all sports bettors. This U. S.-focused study reflects a particular regulatory and cultural environment. Findings may differ in regions and countries with alternative norms or betting policies. Future research should employ longitudinal tracking, behavioral data (e.g., betting app metrics), and theoretical models of moral disengagement, habit formation, and self-regulation. These additions could help illuminate why some fans escalate their betting behaviors while others maintain a critical distance.

## Conclusion

This study enhances our understanding of how sports betting affects fan behavior, emotional responses, and moral perceptions, particularly among NFL audiences. Four contributions stand out. First, engagement is clearly elevated by betting, especially for those who self-identify as obsessed. This aligns with Uses and Gratifications Theory, suggesting that betting boosts emotional stimulation and perceived relevance. Second, moral concern and worry about others do not significantly predict engagement or dependency, though they are moderately associated. This suggests possible emotional dissonance or normalization among fans. Third, dependency is not demographic-driven. It is strongly tied to obsession, which cuts across age and gender categories, implying a deeper psychological pattern rather than an external cause. Finally, moral judgment and action diverge. Those most engaged are least likely to advocate confronting others, possibly due to denial or disengagement mechanisms.

These findings offer value to sports organizations, app developers, and public health stakeholders. While betting enhances revenue and engagement, it also introduces psychological consequences that may undermine well-being. Regulatory frameworks must weigh excitement and immersion against potential harm.

As digital sports ecosystems evolve, understanding and mitigating these cognitive tensions will be critical. This study offers an empirically grounded step in that direction.

## Data Availability

The raw data supporting the conclusions of this article will be made available by the authors, without undue reservation.

## Appendix

Instrument

Using a 1–5 scale of agreement (with 1 = strongly disagree and 5 = strongly agree,) to what extent do you agree or disagree with the following statements?

1. I watch sports more than I did previously because of sports betting.
2. I watch sports I previously would not have watched because of sports betting.
3. I watch games not involving my favorite team because of sports betting.
4. I find myself watching sports games only because of bets.
5. I put more time into sports research outside of watching games because of betting.
6. I consume more sports media because of betting.
7. I consume more sports media than I used to because of betting.
8. I enjoy watching sports less because of sports betting.
9. My investment in my favorite teams has changed because of sports betting.
10. Sports betting has positively influenced coverage of sports on social media.
11. Sports betting has positively influenced coverage of sports in talk shows/ broadcast.
12. Have you placed a bet on a sports game (Not including a party-related bet like squares or bets without any of your money or items of value at stake)? Yes/No
13. (If YES, proceeds to question 13, if NO skips to question 18)

Using the 1–5 scale of agreement, to what extent do you agree or disagree with the following statements?

14. Watching games with the betting connection makes the sport more enjoyable.
15. I only watch games if I am betting on it.
16. I like to place bets on games I do not watch.
17. I feel that I am “missing out” if I do not place a bet on a game I am watching.
18. I feel obsessed with sports betting.
19. YES or NO? I know one or more people who (in your opinion) are obsessed with sports betting. (If YES, proceeds to question 19, if NO skips to question 23)
20. Based on your opinion, approximately how many people do you know that are “obsessed” with sports betting. (A) 1–2 (B) 3–4. (C) 5–6. (D) 7 or more
21. According to AddictionCenter.com, the definition of sports addiction is “Sports betting addiction is a behavioral disorder characterized by a persistent and uncontrollable desire to bet on sports despite adverse outcomes.”
After reading this definition, approximately how many people do you know that (in your opinion) are obsessed/addicted to sports betting?
(A) 1–2 (B) 3–4. (C) 5–6. (D) 7+.
To what extent do you agree or disagree with the following statements
22. I feel concerned about family/friends I consider obsessed with sports betting.
23. I feel it is important for me and others concerned about a family member / friend to confront them about their betting habits.
24. How old are you?
25. What is your gender?
