# Supplementary material for: All bets are on: obsession, engagement, and moral tension in sports betting behavior
Source: Front Psychol. 2025 Jul 14;16:1608414. doi: 10.3389/fpsyg.2025.1608414 (PMC12301884; doi:10.3389/fpsyg.2025.1608414)
Supplement: Supplementary file 1 [file Table_1.docx]

# Table 1. Standardized Regression Coefficients Predicting Bet-Driven Sports Engagement.

| Predictor | β (Standardized) | *p*-value |
| --- | --- | --- |
| Age | -0.144 | .001** |
| Sex assigned at birth (Male = 1, Female = 0) | -0.091 | .039* |
| “I enjoy watching sports less because of sports betting.” | 0.146 | .001** |
| “I am concerned about family/friends I consider obsessed with sports betting.” | 0.107 | .019* |
| “It is important to confront a family member/friend about their betting habits.” | 0.221 | < .001*** |

Note. **p** < .05 (*), **p** < .01 (**), **p** < .001 (***)
